# Testing optimally weighted combination of variants for hypertension

**DOI:** 10.1186/1753-6561-8-S1-S59

**Published:** 2014-06-17

**Authors:** Xingwang Zhao, Qiuying Sha, Shuanglin Zhang, Xuexia Wang

**Affiliations:** 1 Department of Mathematical Sciences, Michigan Technological University, 1400 Townsend Drive, Houghton, MI 49931, USA; 2 Joseph J.Zilber School of Public Health,University of Wisconsin, P.O. Box 413, Milwaukee, WI 53201, USA

## Abstract

Testing rare variants directly is possible with next-generation sequencing technology. In this article, we propose a sliding-window-based optimal-weighted approach to test for the effects of both rare and common variants across the whole genome. We measured the genetic association between a disease and a combination of variants of a single-nucleotide polymorphism window using the newly developed tests TOW and VW-TOW and performed a sliding-window technique to detect disease-susceptible windows. By applying the new approach to unrelated individuals of Genetic Analysis Workshop 18 on replicate 1 chromosome 3, we detected 3 highly susceptible windows across chromosome 3 for diastolic blood pressure and identified 10 of 48,176 windows as the most promising for both diastolic and systolic blood pressure. Seven of 9 top variants influencing diastolic blood pressure and 8 of 9 top variants influencing systolic blood pressure were found in or close to our top 10 windows.

## Background

Hypertension is a common chronic destructive disease with unknown complex etiology [1]. More than1billion people worldwide have hypertension, defined as blood pressure (BP) ≥140 mm Hg systolic (SBP) or ≥90 mm Hg diastolic (DBP) [2], which is a major risk factor for stroke, myocardial infarction, heart failure, and a cause of chronic kidney disease [3-5]. Both genetic and environmental bases are likely to contribute to this disease. Ehret *et al*. conducted a large-scale genome-wide association study of hypertension in 2011 and identified 10 novel loci related to BP physiology [6]. Although numerous common genetic variants with small effects on BP have been identified [6-8], the identified variants account for only a small fraction of disease heritability [9]. One potential source of missing heritability is the contribution of rare variants. Recently, next-generation sequencing technologyhas enabled the sequencing of the whole genome of large groups of individuals,which makes directly testing rare variants feasible. The Genetic Analysis Workshop 18 (GAW18) data, which consists of a whole genome sequencingdata set, is a large-scale pedigree-based sample with 959 individuals, 464 directly sequenced and the rest imputed.

Several statistical methods have been proposed to detect associations of rare variants, including the combined multivariate and collapsing (CMC) method [10] and the weighted sum statistic (WSS) [11]. We have proposed a novel test for measuringthe effect of an optimally weighted combination of variants (TOW) [12]. In addition, based on the TOW, we proposed a variable weight-TOW (VW-TOW) aiming to test effects of both rare and common variants. Both TOW and VW-TOW are applicable to quantitative and qualitative traits, allow covariates, and are robust to directions of effects of causal variants.

In this article, we report a novel whole genome sliding window approach to detect genetic association between a trait and single-nucleotide polymorphism (SNP) regions across the entire genome. This approach integrates TOW and VW-TOW with the concept of sliding window [13]. Applied to the GAW18 replication 1, chromosome 3 data set, our approach yielded results consistent with the top genes influencing simulated SBP and DBP, which were generated from the GAW18 simulation model.

## Methods

Consider a sample of *n *individuals. Each individual has been genotyped at *M *variants in a genomic region. Denote $y_{i}$ as the quantitative trait value. Denote $X_{i}={\left(x_{i1},...,x_{iM}\right)}^{T}$ as the genotypic score of the *i*^th ^individual, where $x_{im}\in \left\{0,1,2\right\}$ is the number of minor alleles that the *i*^th ^individual has at the *m*^th ^variant.

Suppose we have *p *covariates. Let ${\left(z_{i1},...,z_{ip}\right)}^{T}$ denote covariates of the *i*^th ^individual. We adjust both trait value $y_{i}$ and genotypic score $x_{im}$ for the covariates by applying linear regressions. That is, $y_{i}=\alpha_{0}+\alpha_{1}z_{i1}+...+\alpha_{p}z_{ip}+\epsilon_{i}$ and $x_{im}=\alpha_{0m}+\alpha_{1m}z_{i1}+...+\alpha_{pm}z_{ip}+\tau_{im}$.

Let ${ỹ}_{i}$ and ${\tilde{x}}_{im}$ denote the residuals of $y_{i}$ and $x_{im}$, respectively. Denote ${\tilde{X}}_{i}=\left({\tilde{x}}_{i1},\ldots ,{\tilde{x}}_{iM}\right)$ as the residuals of the genotypic score of the $i^{th}$ individual.

Using the generalized linear model (GLM) to model the relationship between trait values and genotypes is equivalent to modeling the relationship between the residuals of trait values and the residuals of genotypes through GLM (1), where g() is a monotone "link" function.

(1) $$g\left(E\left({ỹ}_{i}|{\tilde{X}}_{i}\right)\right)=\beta_{0}+\beta_{1}{\tilde{x}}_{i1}+\cdots +\beta_{M}{\tilde{x}}_{iM}$$

Under the GLM, the score test statistic to test the null hypothesis $H_{0}:\beta =0$ is given by $S=U^{T}V^{-1}U$, where $U=\sum_{i=1}^{n}\left({ỹ}_{i}-\bar{ỹ}\right)\left({\tilde{X}}_{i}-\bar{\tilde{X}}\right)$ and $V=\frac{1}{n}\sum_{i=1}^{n}{\left({ỹ}_{i}-\bar{ỹ}\right)}^{2}\sum_{i=1}^{n}\left({\tilde{X}}_{i}-\bar{\tilde{X}}\right){\left({\tilde{X}}_{i}-\bar{\tilde{X}}\right)}^{T}$. The statistic *S *asymptotically follows a chi-square distribution with k=rank(V) degrees of freedom (DF). For rare variants, however, the score test may lose power as a result of the sparse data and a large DF*k*. In rare variants association studies, to test for the effect of the weighted combination of variants, $x_{i}=\overset{M}{\underset{m=1}{\sum }}w_{m}x_{im}$, the score test statistic becomes

$$S\left(w_{1},\cdots \;,w_{M}\right)=n\frac{(\sum_{i=1}^{n}\left({ỹ}_{i}-\bar{ỹ}\right)\left({\tilde{x}}_{i}-\bar{\tilde{x}}\right)){\;}^{2}}{\sum_{i=1}^{n}{\left({ỹ}_{i}-\bar{ỹ}\right)}^{2}\sum_{i=1}^{n}{\left({\tilde{x}}_{i}-\bar{\tilde{x}}\right)}^{2}}=n\frac{(\sum_{m=1}^{M}w_{m}\sum_{i=1}^{n}\left({ỹ}_{i}-\bar{ỹ}\right)\left({\tilde{x}}_{im}-{\bar{\tilde{x}}}_{m}\right)){\;}^{2}}{\sum_{i=1}^{n}{\left({ỹ}_{i}-\bar{ỹ}\right)}^{2}\sum_{i=1}^{n}{\left({\tilde{x}}_{i}-\bar{\tilde{x}}\right)}^{2}}.$$

Because rare variants are essentially independent, we have

$$\sum_{i=1}^{n}{\left({\tilde{x}}_{i}-\bar{\tilde{x}}\right)}^{2}={\sum_{m=1}^{M}\sum_{m=1}^{M}w_{m}w_{l}\sum_{i=1}^{n}\left({\tilde{x}}_{im}-{\bar{\tilde{x}}}_{m}\right)\left({\tilde{x}}_{il}-{\bar{\tilde{x}}}_{l}\right)\approx \sum_{m=1}^{M}w_{m}^{2}\sum_{i=1}^{n}\left({\tilde{x}}_{im}-{\bar{\tilde{x}}}_{m}\right)}^{2}$$

Let $a_{m}=\frac{\sum_{i=1}^{n}\left({ỹ}_{i}-\bar{ỹ}\right)\left(x_{im}-\bar{\tilde{x}}\right)}{\sqrt{\sum_{i=1}^{n}{\left(x_{im}-{\bar{\tilde{x}}}_{m}\right)}^{2}}}$ and $u_{m}=w_{m}\sqrt{\sum_{i=1}^{n}{\left({\tilde{x}}_{im}-{\bar{\tilde{x}}}_{m}\right)}^{2}}$.

Then, the score test statistic is approximately equal to $S_{0}\left(w_{1},\cdots \;,w_{M}\right)=n\frac{{\left(\sum_{m=1}^{M}a_{m}u_{m}\right)}^{2}}{\sum_{i=1}^{n}{\left({ỹ}_{i}-\bar{ỹ}\right)}^{2}\sum_{m=1}^{M}u_{m}^{2}}.$

As a function of $\left(u_{1},\cdots \;,u_{M}\right)$, $S_{0}\left(w_{1},\cdots w_{M}\right)$ reaches its maximum when $u_{m}=a_{m}$ or $w_{m}=\sum_{i=1}^{n}\left({ỹ}_{i}-\bar{ỹ}\right)\left({\tilde{x}}_{im}-{\bar{\tilde{x}}}_{m}\right)/\sum_{i=1}^{n}{\left({\tilde{x}}_{im}-{\bar{\tilde{x}}}_{m}\right)}^{2}$(m=1,⋯,M). We denote $w_{m}^{o}$ as the optimal weight which is given by $w_{m}^{o}=\sum_{i=1}^{n}\left({ỹ}_{i}-\bar{ỹ}\right)\left({\tilde{x}}_{im}-{\bar{\tilde{x}}}_{m}\right)/\sum_{i=1}^{n}{\left({\tilde{x}}_{im}-{\bar{\tilde{x}}}_{m}\right)}^{2}$. Let ${\tilde{x}}_{i}^{o}=\sum_{m-1}^{M}w_{m}^{o}{\tilde{x}}_{im}$. Then $S_{0}\left(w_{1}^{o},\cdots \;,w_{M}^{o}\right)=n\sum_{i=1}^{n}\left({ỹ}_{i}-\bar{ỹ}\right)\left({\tilde{x}}_{1}^{o}-{\bar{\tilde{x}}}^{o}\right)/\sum_{i=1}^{n}{\left({ỹ}_{i}-\bar{ỹ}\right)}^{2}.$ We propose the new test statistic TOW to test the effect of the optimally weighted combination of variants $\sum_{m=1}^{M}w_{m}^{o}{\tilde{x}}_{im}$ as $T_{T}=\sum_{i=1}^{n}\left({ỹ}_{i}-\bar{ỹ}\right)\left({\tilde{x}}_{1}^{o}-{\bar{\tilde{x}}}^{o}\right)$. $T_{T}$ is equivalent to $S_{0}\left(w_{1}^{o},\cdots w_{M}^{o}\right)$ since $\sum_{i=1}^{n}{\left({ỹ}_{i}-\bar{ỹ}\right)}^{2}$ is a constant. The optimal weight $w_{m}^{o}$ will put big weights to the variants that have strong associations with the traits of interest and adjust the direction of the association. Also, $w_{m}^{o}$ will put big weights to rare variants. TOW targets rare variants and will lose power when testing for the effect of both rare and common variants. For testing the effects of both rare and common variants, we propose a new statistic, VW-TOW. We divide variants into rare (minor allele frequency [MAF] <the rare variant threshold [RVT]) and common (MAF > RVT), and apply TOW to the rare and common variants separately.

Define the test statistic of VW-TOW as $T_{VW-T}={\text{min}}_{0\leq \lambda \leq 1}p_{\lambda }$, where $p_{\lambda }$ is the *p *value of $T_{\lambda }$.$T_{\lambda }=\lambda \frac{T_{r}}{\sqrt{var\left(T_{r}\right)}}+\left(1-\lambda \right)\frac{T_{c}}{\sqrt{var\left(T_{c}\right)}}$, $T_{r}$ and $T_{c}$ denote the test statistics of TOW for rare and common variants, respectively. Here, we evaluate the minimization by dividing the interval [0, 1] into *K *subintervals of equal-length. Let $\lambda_{k}=k/K$ for k=0,1,⋯,K. Then, ${\text{min}}_{0\leq \lambda \leq 1}p_{\lambda }={\text{min}}_{0\leq k\leq K}p_{\lambda_{k}}$.

We use permutation tests to evaluate *p *values of both $T_{T}$ and $T_{VW-T}$. To evaluate the *p *value of the test $T_{T}$, let $T_{T}^{0}$ denote the value of the test statistic based on the original data set. For each permutation, we randomly resample from residuals of trait values and denote the value of the test statistic based on the permuted data setby $T_{T}^{per}$. We perform the permutation procedure many times. Then the *p *value of the test is the proportion of the number of permutations with $T_{T}^{per}>T_{T}^{0}$. We permute *B *times of permutations to evaluate the *p *value of $T_{VW-T}$. Let $T_{r}^{\left(b\right)}$ and $T_{c}^{\left(b\right)}$ denote the values of $T_{r}$ and $T_{c}$ based on the $b^{th}$ permuted data, where b=0 represents the original data. Based on $T_{r}^{\left(b\right)}$ and $T_{c}^{\left(b\right)}$(b=0,1,⋯,B), we can calculate $T_{\lambda_{k}}^{b}$ for b=0,1,⋯,B and k=0,1,⋯,K, where $\text{var}\;\left(T_{r}\right)$ and $\text{var}\;\left(T_{c}\right)$ are estimated using $T_{r}^{\left(b\right)}$ and $T_{c}^{\left(b\right)}$(b=1,⋯,B). Then, we transfer $T_{\lambda_{k}}^{\left(b\right)}$ to $p_{\lambda_{k}}^{\left(b\right)}$ by $p_{\lambda_{k}}^{\left(b\right)}=\frac{\sum_{i=0}^{B}I\left(T_{\lambda_{k}}^{\left(i\right)}>T_{\lambda_{k}}^{\left(b\right)}\right)}{B}$, where I() is the indicator function. Let $p^{\left(b\right)}={\text{min}}_{0\leq k\leq K}p_{\lambda_{k}}^{\left(b\right)}$. Then the *p *value of $T_{VW-T}$ is given by $\frac{\sum_{i=1}^{B}I\left(p^{\left(b\right)}<p^{\left(0\right)}\right)}{B}$, where I() is the indicator function.

We use TOW and VW-TOW to analyze the data set of unrelated individuals of GAW18 replication 1 on chromosome 3. To apply TOW and VW-TOW to the entire chromosome 3, we propose a sliding-window approach [13]. To use sliding windows, we divide all SNPs into contiguous windows and apply TOW and VW-TOW in each window. Suppose that we use windows with a window size of S, then, all the SNPs can be divided into windows: 1 to S, S+1 to 2S, 2S+1 to 3S, and so on.

To analyze the data set of GAW18 replication 1, chromosome 3 for unrelated individuals, we set the window size as 20. First we performed quality control tests for the genotype data with the PLINK toolset. We used 10,000,000 permutations to evaluate the empirical *p *values of TOW for DBP and SBP data, and 100,000 permutations to evaluate the empirical *p *values of VW-TOW for DBP and SBP data. Becausethe sample of unrelated individuals in GAW18 is relatively small, it is not so reasonable to claim the significance either by the false-discovery rate or by the Bonferroni-corrected threshold. Therefore, we recommend the top 10 most promising windows with the smallest *p *values for follow-up studies.

## Results

We applied TOW and VW-TOW incorporating the sliding window approaches to analyze the hypertension unrelated individuals'data set of GAW18. To facilitate comparisons among GAW18 contributions, we analyzed only replicate 1 on chromosome 3. To evaluate type I error rates of TOW and VW-TOW, we used all 200 replicates of simulated phenotype data. There are 157 unrelated individuals in the GAW18 pedigree-based sample. Among the 157 individuals, 142 have observations for SBP, DBP, and other demographic/clinical variables at exam 1. Our analysis was based on the 142 individuals and their genotypes, quantitative trait SBP, DBP, and other characteristicsat exam 1.

The total genotyping rate in the 142 individuals is 0.9997. We did not find any duplicated samples or sample contamination. No individual was filtered out from the multidimensional scaling (MDS) analysis. Of the 1,215,399 SNPs on chromosome 3, we removed 251,892 completely missing SNPs and retained 963,507 SNPs for final analysis. Because SBP and DBP varied by sex and increased with age, age and sex were considered as covariates in this study.

We listed the top 10 most promising windows out of 48,176 windows across the entire chromosome 3. The top 8 windows all reside in gene *MAP4*, which is the most susceptible gene on chromosome 3 for hypertension. Seven of 9 top variants influencing DBP and 8 of 9 top variants influencing SBP on chromosome 3 were found in or close to our top windows. Tables 1 and 2 show the top 10 most promising windows by TOW that are associated with DBP and SBP, respectively. The *p *values of TOW in the top 3 windows of Table 1 are very small. SNP 3_47957996, 3_ 47956424, and 3_47957741 are the third, fourth, and ninth variants in Table 2 of the GAW18 answer sheet. They all fell into our third window in Table 1 and the first window in Table 2.

**Table 1:** Top 10 most promising windows associated with DBP

WID	Chr	Physical location	Empirical p_TOW_	Empirical p_VW-TOW_	Gene	Reference variants
1	3	48117215,48121372	2.34 × 10^−7^	0.0005	*MAP4*	
2	3	48063171,48068858	4.95 × 10^−7^	0.0005	*MAP4*	
3	3	47957289,47961091	4.09 × 10^−6^	0.0006	*MAP4*	3_47957996 3_47956424 3_47957741
4	3	48034051,48040240	1.42 × 10^−5^	0.001	*MAP4*	3_48040284 3_48040283
5	3	48089115,48094079	2.06 × 10^−5^	0.001	*MAP4*	
6	3	48005035,48009105	2.69 × 10^−5^	0.0015	*MAP4*	
7	3	47929938,47935009	5.29 × 10^−5^	0.001	*MAP4*	
8	3	47912703,47920240	9.06 × 10^−5^	0.001	*MAP4*	3_47913455
9	3	4474736,4477687	0.036	0.071	*SUMF1*	3_45008742
10	3	56871312,56875674	0.03	0.058	*ARHGF3*	3_56870810*

*Chr*, Chromosome; *empirical p_TOW _*, *p *value of TOW; *empirical p_VW-TOW_*, *p *value of VW-TOW; *reference variants*, top 55 variants influencing SBP and DBP in Table 2 of the answers of GAW18; *WID*, window ID.

*The variants are provided in the Supplemental Table 1 of the answer sheet of GAW18.

**Table 2:** Top 10 most promising windows associated with SBP

WID	Chr	Physical location	Empirical p_TOW_	Empirical p_VW-TOW_	Gene	Reference variants
1	3	47957289,47961091	0.005	0.004	*MAP4*	3_47957996 3_47956424 3_47957741
2	3	48034051,48040240	0.003	0.007	*MAP4*	
3	3	47990787,47999337	0.01	0.013	*MAP4*	
4	3	48040283,48046708	0.02	0.01	*MAP4*	3_48040284 3_48040283
5	3	47912703,47920240	0.03	0.017	*MAP4*	3_47913455
6	3	48121395,48126740	0.015	0.032	*MAP4*	
7	3	47929938,47935009	0.025	0.04	*MAP4*	
8	3	48063171,48068858	0.015	0.01	*MAP4*	
9	3	58104877,58108614	0.01	0.031	*FLNB*	3_58109162
10	3	15664089,15667215	0.039	0.011	*BTD*	3_15686693

*Chr*, Chromosome; *empirical p_TOW _*, *p *value of TOW; *empirical p_VW-TOW_*, *p *value of VW-TOW; *reference variants*, top 55 variants influencing SBP and DBP in Table 2 of the answers of GAW18; *WID*, window ID.

**Figure 1:**
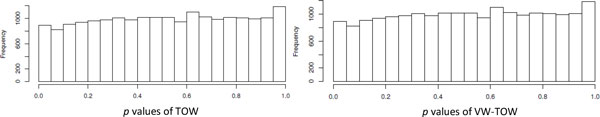
**Histograms of *p *values for TOW and VW-TOW**.

To evaluate the type I error rates of the proposed sliding window approach, we chose 100 blocks (20 variants in each block) from chromosome 3 that are far from causal variants. In each block, we applied TOW and VW-TOW to each of the 200 replicates to test association between genotypes and the trait DBP. We obtained 1 *p *value for each replicate and each block. Figure 1 shows the histograms of TOW and VW-TOW. The histograms indicate that the type I error rates of both TOW and VW-TOW are under control.

## Discussion

In this article, we proposed a sliding-window-based optimal weighted approach to test for the effects of both rare and common variants across the whole genome. In each window, our recently developed TOW and VW-TOW were applied to test genetic association between a disease and a combination of variants. Then, we applied the method to unrelated individuals of GAW18 on replicate 1, chromosome 3. We detected 3 susceptible windows across chromosome 3 for DBP and identified 10 out of 48,176 windows as the most promising windows for DBP and SBP. Becausethis is a simulated dataset, it is possible that the other genes identified were not listed in the top 10 windows but are actually related to SBP or DBP.

In this study, we use each window of size 20 across the entire chromosome 3. How to choose an appropriate window size is a critical question. We evaluated the effect of window size by running window sizes at 30, 40, and 50, respectively. However, the power of TOW was not increased when using a larger window size. Although the power of VW-TOW was slightly increased when using a larger window size, no window can pass the entire chromosome 3 Bonferroni-corrected threshold.

TOW and VW-TOW can be robust to population stratification by adjusting the first *K *principalcomponents (PCs) of genotypes at genomic markers as covariates when calculating the residuals of trait and of genotype matrix. In this GAW18 data analysis, we did not adjust for PCsbecausewe believed that population stratification was not severe in this data based on our MDS analysis.

**Figure 2:**
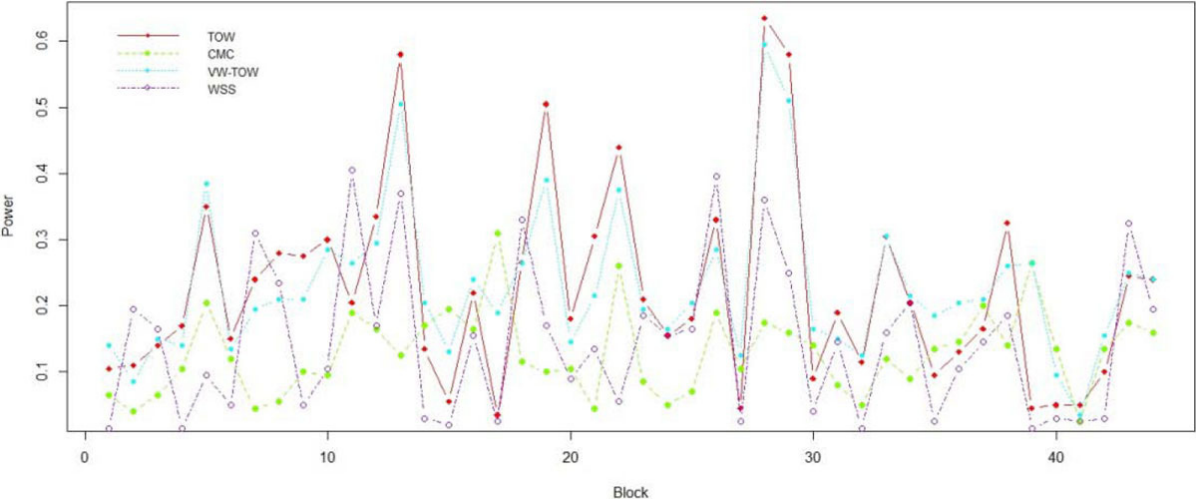
**Power comparisons of TOW, CMC, VW-TOW, and WSS using DBP as phenotype measurement**. The numbers on the x axis refer to the 44 blocks of gene *MAP4.*

To further assess our new approach, we compared the power of TOW, VW-TOW, CMC, and WSS to detect association between gene *MAP4 *and DBP. The *MAP4 *was split into 44 windows (blocks) with 20 variants in each window. In each window, we calculated the power of each method based on 200 replicates. The power comparisons based on phenotype measurement DBP are given in Figure 2. This figure shows that in most of the windows, TOW is the most powerful test; VW-TOW is the second most powerful test.

## Competing interests

The authors declare that they have no competing interests.

## Authors' contributions

XW designed the overall study. XZ and XW conducted statistical analysis. XZ, QS, and SZ drafted the manuscript. All authors read and approved the final manuscript.
